# Pathophysiology and treatment of focal segmental glomerulosclerosis: the role of animal models

**DOI:** 10.1186/1471-2369-14-74

**Published:** 2013-04-01

**Authors:** Sylvana ML de Mik, Martin J Hoogduijn, Ron W de Bruin, Frank JMF Dor

**Affiliations:** 1 Laboratory of experimental surgery, Department of Surgery, Erasmus MC, University Medical Center, Rotterdam, The Netherlands; 2 Department of surgery, Erasmus MC, University Medical Center, Rotterdam, The Netherlands; 3 Department of Internal Medicine, Erasmus MC, University Medical Center Rotterdam, Rotterdam, The Netherlands

**Keywords:** Focal segmental glomerulosclerosis, Animal model, Remnant kidney, Adriamycin, Puromycin aminonucleoside-induced nephrosis, hiv, Mpv-17, α-actinin 4

## Abstract

Focal segmental glomerulosclerosis (FSGS) is a kidney disease with progressive glomerular scarring and a clinical presentation of nephrotic syndrome. FSGS is a common primary glomerular disorder that causes renal dysfunction which progresses slowly over time to end-stage renal disease. Most cases of FSGS are idiopathic Although kidney transplantation is a potentially curative treatment, 40% of patients have recurrence of FSGS after transplantation. In this review a brief summary of the pathogenesis causing FSGS in humans is given, and a variety of animal models used to study FSGS is discussed. These animal models include the reduction of renal mass by resecting 5/6 of the kidney, reduction of renal mass due to systemic diseases such as hypertension, hyperlipidemia or SLE, drug-induced FSGS using adriamycin, puromycin or streptozotocin, virus-induced FSGS, genetically-induced FSGS such as via Mpv-17 inactivation and α-actinin 4 and podocin knockouts, and a model for circulating permeability factors. In addition, an animal model that spontaneously develops FSGS is discussed. To date, there is no exact understanding of the pathogenesis of idiopathic FSGS, and there is no definite curative treatment. One requirement facilitating FSGS research is an animal model that resembles human FSGS. Most animal models induce secondary forms of FSGS in an acute manner. The ideal animal model for primary FSGS, however, should mimic the human primary form in that it develops spontaneously and has a slow chronic progression. Such models are currently not available. We conclude that there is a need for a better animal model to investigate the pathogenesis and potential treatment options of FSGS.

## Background

Focal segmental glomerulosclerosis (FSGS) is a disease with progressive glomerular scarring. Studies [1,2] have shown that podocytes are the main cells involved in the development of FSGS. Podocytes are epithelial cells of the visceral layer of the kidney’s Bowman’s capsule. Their function is to form a filtration structure that prevents protein loss to the urine. Destruction of podocytes induced by cellular stress results in sclerosis of part (segmental) of the glomerular capillaries in a minority (focal) of glomeruli. If the sclerosis continues, global glomerulosclerosis will develop. Clinically, the loss of podocytes and their filtration function results in nephrotic syndrome, consisting of proteinuria, hypoalbuminemia, hypercholesterolemia and peripheral edema [3].

FSGS has an incidence of 7 per million, and in 20% of children and in 40% of adults, it is the underlying cause of nephrotic syndrome. When FSGS is accompanied by high proteinuria levels at the time of presentation, 50% of cases progress to end-stage renal disease (ESRD) within 3 to 8 years, making FSGS causal for 4% of all ESRD cases [3]. After kidney transplantation (KT) for primary FSGS, the recurrence rate is 40% [4,5].

In 80% of FSGS patients, the etiology is unknown. In the remaining 20% of cases, FSGS develops secondary to other underlying diseases. Genetic causes (α-actinin 4 mutations) were found, as well as forms that were HIV virus-associated, induced by drugs such as interferon-γ, caused by a congenital reduction in renal mass (unilateral agenesis) or by reduction of renal mass due to systemic disease such as hypertension [6].

Ever since FSGS was first described by Arnold Rich in 1959 [7], many studies have been conducted to understand its pathogenesis and to identify risk factors and/or possible treatments for this disease. In order to facilitate the study of FSGS, different kinds of animal models have been developed to mimic the clinical pathological features of human FSGS. In this review, we briefly summarize the pathogenesis causing FSGS in humans, and subsequently provide an overview of the different animal models that are currently being used or have been used in the past to investigate FSGS.

## Pathogenesis of FSGS in humans

### Secondary FSGS caused by systemic diseases

FSGS caused by systemic diseases such as hypertension, diabetes and obesity will eventually lead to glomerular hyperfiltration-hypertension, which causes mechanical stress on the podocytes [8]. Mechanical stress leads to structural changes in podocyte cell bodies and reinforces their adherence to the glomerular basement membrane (GBM). These changes are facilitated by angiotensin II (Ang II)- and transforming growth factor beta (TGF-β) dependent pathways. Ang II activation leads to generation of reactive oxygen species, rearrangement of slit diaphragm proteins, down regulation of nephrin expression and podocyte hypertrophy. In addition, Ang II activates VEGF, which leads to an increase in albumin uptake due to angiogenesis and increased permeability of the capillary endothelium for albumin. TGF-β down regulates α-3β-1 integrin expression, which leads to foot process effacement and affects the ability of podocytes to adhere to the GBM. Podocyte hypertrophy, foot process effacement and detachment from GBM eventually lead to apoptosis and podocyte depletion. Diabetes can also directly activate the Ang II- and TGF-β dependent pathways [8].

### Drug-induced FSGS

Several drugs are able to induce FSGS. Pamidronate is used for the treatment of myeloma and metastatic cancers. It has an inhibitory effect on actin cytoskeleton formation in osteoclasts, and may have a similar effect on the podocyte cytoskeleton. IFN-γ can affect podocytes through the IFN-α and IFN-β receptors on podocytes. In the transplanted kidney, FSGS may be induced by the toxic effects of immunosuppressive drugs. For instance, Rapamycin can induce FSGS by reducing the expression of Nephrin and other proteins composing the slit diaphragm and cytoskeleton [9].

### Virus-induced FSGS

Virus-induced FSGS can be caused by direct infection of the podocyte or by the release of inflammatory cytokines by other virus-infected cells that interact with podocyte receptors. HIV-1 directly infects the podocytes and tubular epithelial cells. Viral genes such as Negative regulatory factor (Nef) and Viral protein R (Vpr) are causal for HIV-associated nephropathy (HIVAN). Nef promotes podocyte dedifferentiation and proliferation and dysregulation of actin cytoskeleton. Vpr mediates tubular epithelial G2 cell-cycle arrest and apoptosis. HIVAN causes the collapsing variant of FSGS [3].

### Genetic defects causing FSGS

**Figure 1:**
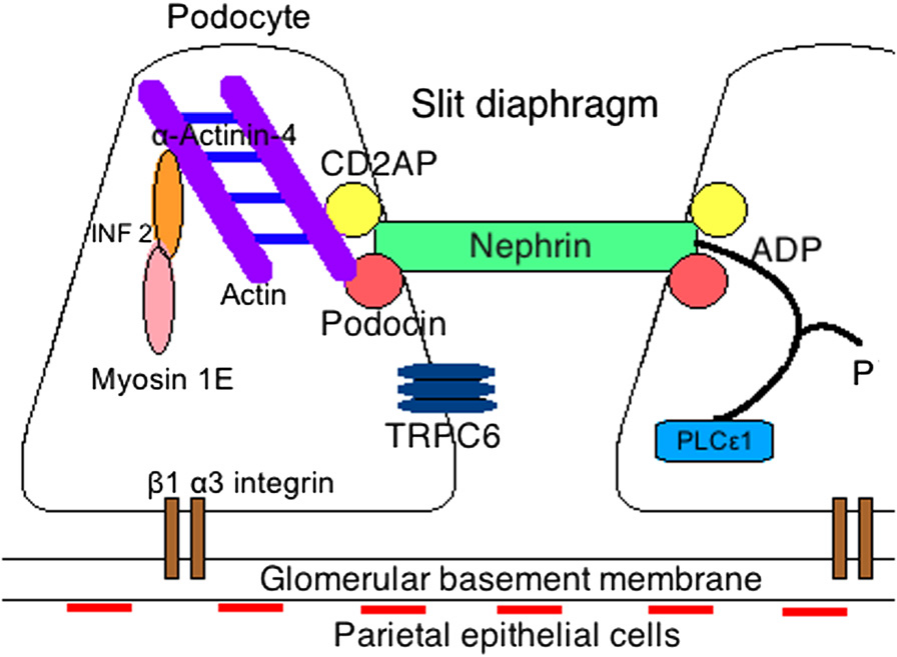
Regulators of the podocyte actin cytoskeleton and the slit diaphragm.

In familial FSGS several genetic defects have been discovered, which account for 8% of FSGS causes [10]. The affected genes encode important regulators of the actin cytoskeleton of podocytes and form an extensive list of factors. It includes Nephrin, a transmembrane protein and an important structure of the slit diaphragm. Through its cytoplasmic domain it regulates podocyte actin dynamics [11]. Podocin is an adapter protein facilitating a functioning filtration barrier by directing nephrin and CD2AP to the right location in the slit diaphragm and therefore connecting the slit diaphragm to the actin cytoskeleton [12]. Besides being part of this connection, CD2AP also helps maintaining the organization of the podocyte actin cytoskeleton through regulation of cytosolic cathepsin L expression [13]. α-Actinin-4 is an actin-binding protein necessary for accurate regulation of actin turnover, important for maintaining a normal morphology and function of podocytes [14]. Transient receptor potential cation channel 6 (TRPC6) is a calcium channel that is likely to have an effect on the actin cytoskeleton through the RhoA-pathway, a known modulator of the actin cytoskeleton [15]. In addition, TRPC6 combined with podocin is thought to act as a mechanosensor at the slit diaphragm by translating mechanical tension to ion-channel activity [16]. Phospholipase C (PLC) ε1 is important for the development and dedifferentiation of podocytes and interaction with nephrin through GTPase-activating protein 1. Its precise function in mature podocytes is however unknown [17]. Inverted formin (INF) 2 is part of the actin-regulating proteins inhibiting actin polymerization via Rho/mDia-pathway [18]. Myosin 1^E^, a molecular motor translocating cargo proteins along actin filaments, is important for podocyte migration and may also stabilize the podocyte cytoskeleton [19]. ARHGAP24 is a gene necessary for a normal functioning Rho-pathway, which, as mentioned before, is an important regulator of the actin cytoskeleton [20] (Figure 1). For a more detailed description of these genetic defects, we recommend the following reference [21].

### Susceptibility genes

In addition to gene defects that have an immediate effect on the podocyte cytoskeleton, polymorphisms in Myosin heavy chain 9 (MYH9) and Apolipoprotein L1 (APOL1) genes were identified that affect the susceptibility of podocytes to mechanical stress. Mechanical stress causes reorganization of the actin skeleton by replacing transversal stress fibers with radial stress fibers connected to an actin-rich centre [22]. It was found that hypertension associated end-stage renal disease in African-Americans is substantially related to both polymorphisms. MYH9 interacts statically with F-actin to maintain membrane tension and cell shape and is therefore a likely candidate to play a role in the development of FSGS. APOL1, however, has a stronger association with FSGS than MYH9 and could possibly be the actual susceptibility gene [23].

### Circulating permeability factors

The first speculations about the excistence of circulating permeabillty factors date from 1972. Three cases of patients with steroid-resistant nephrotic syndrome were described that progressed to ESRD within 2 to 6 years. After receiving KT all 3 patients showed a recurrence of nephrotic syndrome within 1 to 5 months. The kidneys of 2 patients who died from immunological causes, were studied. Both the native kidney as well as the allograft showed FSGS in the juxtamedullary glomeruli, suggesting a recurrence of the original disease in the transplanted kidney. To explain these observations it was hypothesized that systemic circulating factors might be involved [24]. Recently, a case was reported in which a primary FSGS patient that received a kidney transplant developed marked proteinuria on the second day post transplantation. A biopsy demonstrated recurrence of the disease despite plasmapheresis. On day 14 the allograft was removed due to worsening of the disease. The removed kidney was then retranspanted into a patient with ESRD caused by type 2 diabetes. After transplantation the kidney regained function and biopsy analysis showed a reversal of the histopathological lesions. This case supports the circulating permeability factor theory and that podocyte injury may be reversible before scar formation occurs [25]. In a different study researchers observed that there was an increase in glomerular permeability for albumin when rat glomeruli were exposed to serum samples from FSGS patients. This effect was even more pronounced when serum was used from patients with recurrent disease after transplantation. In addition, glomeruli were exposed to serum from patients with recurrent FSGS after plasmapheresis as well as the plasmapheresis fluid that was gained from these patients. The serum reduced proteinuria whereas the plasmapheresis fluid showed an increase. These findings proved the circulating factor to be primarily confined to the plasma space and not rapidly synthesized after plasmapheresis [26]. The most likely candidate representing the responsible circulating factor is soluble urokinase-type plasminogen activator receptor (suPAR). Significantly elevated suPAR concentrations are observed in patients with primary and recurrent FSGS. High pre-KT suPAR concentrations significantly increase the risk for recurrence of FSGS in the allograft. 1 year post-KT elevated concentrations are observed in patients with recurrence of FSGS compared to non-recurrence in patients. Physiologically low concentrations of suPAR are involved in neutrophil trafficking and stem cell mobilization. In podocytes high concentrations of suPAR can pathologically activate β3 integrin. β3 integrin plays an important role in anchoring podocytes to the glomerular basement membrane and the development of mature foot processes. Activation leads to foot process effacement, proteinuria and initiation of FSGS. SuPAR concentrations can be lowered by plasmapheresis, which decreases β3 integrin activity. This study shows suPAR to be a circulating factor that can cause primary and recurrent FSGS and an important measurable risk factor for recurrent disease [27]. Another possible circulating factor is Cardiotrophin-like cytokine 1, however this candidate awaits further study [3].

## Animal models for FSGS

As described above, the various suggested causes for FSGS in humans all target podocytes. Damaging of podocytes leads to foot process effacement and eventually detachment from the GBM. Adhesions are formed between the denuded GBM and Bowman’s capsule, and parietal epithelial cells (PEC) start producing extracellular matrix (ECM), which causes the typical FSGS lesions [3]. Animal models used to investigate FSGS all induce damage to podocytes and thereby mimic human FSGS.

### Remnant kidney model

The most frequently used animal model for FSGS is the reduced or remnant kidney model in rats. In this model, 4/6 or 5/6 of renal mass is removed by surgically resecting one kidney and ligation of renal artery branches or polectomies to reduce one or two thirds of the renal mass in the contralateral kidney [28]. Most studies use the 5/6 ablation model, since it induces hypertension, pronounced renal damage and FSGS. The 4/6 renal mass reduction model is used as a milder variant since it does not induce hypertension, and only moderate renal dysfunction and glomerulosclerosis [29,30].

To compensate for the loss of renal mass, tubular and glomerular growth occurs. Glomerular growth is achieved by both hyperplasia and hypertrophy. Podocyte growth is structurally slower, as it occurs only through hypertrophy. Therefore, both the capillary and filtration area for a single podocyte is dramatically enlarged. As a consequence, the filtrate cannot be filtered into the urinary space fast enough, causing blockages which divert the filtrate into the space between the podocyte body and foot processes. These maladaptive changes eventually lead to cell destruction and adhesions between the GBM and Bowman’s capsule leading to sclerosis [31]. In addition, studies using polectomy models show only moderate hypertension and slow development of glomerulosclerosis. This is in contrast with ligation models which cause more pronounced hypertension. The presence of hypertension and a rapid development of glomerulosclerosis is caused by the marked up-regulation of components of the renin angiotensin system, namely Ang II, in the inflamed peri-infarct zone in ligation models, leading to structural changes in podocytes [32]. The remnant kidney model can cause podocyte damage through both hyperfiltration-hypertension and via the Ang II pathway, similar to what is seen in human FSGS.

Most rat strains are susceptible to induction of FSGS via the remnant kidney model. Munich-Wistar rats have the benefit of having surface glomeruli that can be used for direct measurement of hemodynamic factors. In contrast, most mouse strains, including C57BL/6, are resistant to the development of FSGS via the remnant kidney model. 129Sv mice are susceptible but the anatomic distribution of the renal artery branches in mice makes it difficult to achieve reproducible 5/6 nephrectomy [28].

There also seems to be a gender-dependent difference in the susceptibility to FSGS. Studies using the remnant kidney model in Munich-Wistar rats and Sprague–Dawley rats have shown that estrogens, mainly estradiol, can protect against FSGS development [33,34].

Studies using the remnant kidney model are conducted for the development of preventive treatment strategies as well as for gaining more insight in underlying pathologies. Using this model it was found that inhibition of thromboxane synthesis [35], the administration of clofibric acid (lipid-lowering agent) [36], troglitazone (peroxisome proliferator-activated receptor-gamma agonist) [37] and Tranilast (antifibrotic agent) [38] can all ameliorate progressive glomerulosclerosis. These studies [35]- [38] all used Sprague–Dawley rats of either male or female sex and reduced renal mass via the renal artery branch ligation technique. Other studies show that absence of functional p21(WAF1/CIP1) in the 129/Sv mouse strain can reduce progression to chronic renal failure [39] and that apolipoprotein E knockout mice do not have an increase in renal injury after subtotal nephrectomy in the presence of hyperlipidemia [40], suggesting a role for this protein in the development of secondary FSGS. Both studies [39,40] used polectomies to induce the remnant kidney model.

The remnant kidney model is limited in its abilities to mimic human FSGS, since the damage is induced via an acute procedure, whereas in human FSGS the damage is induced much slower. However, the remnant kidney model can be used in combination with other FSGS inducing modalities, such as injections with puromycin or with induced hypertension. These FSGS models will be discussed in the next paragraphs.

### Renal mass reduction due to systemic disease

The reduction of renal mass is a secondary event to certain pathologies. In a number of animal models, the decrease in renal mass is the result of chronic damage to the glomerular vessels due to hypertension. In these models, FSGS develops in a similar fashion as in the remnant kidney model, where a decrease in renal mass leads to the availability of a reduced number of glomeruli to filtrate the same amount of serum. Techniques to study hypertension include the use of Sabra hypertension prone rats, which are salt-sensitive animals that develop hypertension when chow and tap water are loaded with 8% NaCl [41]. Renal hypertension can furthermore be caused by administration of norepinephrine (NE) or Ang II. In this model Male Sprague- Dawley rats are used, which are given NE and Ang II intravenously for 14 days, while an inflatable vascular occluder maintains renal perfusion pressure to the left kidney at baseline levels, and exposes the right kidney to elevated perfusion pressure [42]. In addition, hyperlipidemia and obesity models such as Zucker rats have been investigated [43] as well as the ageing, the nephron deficient Munich-Wistar Frömter rat [44].

Besides observing the effect of hypertension on the development of glomerulosclerosis in these two animal models, the Zucker rats show that early influx of glomerular macrophages precedes glomerulosclerosis [43]. The aging Munich-Wistar rats show that age-dependent glomerulosclerosis is reversed after endothelin-1 inhibition. Endothelin-1 seems to have an inhibitory effect on podocyte cell-cycle activity and dedifferentiation. When administering an endothelin-1 antagonist podocytes may re-enter the cell-cycle and recover from previous and age-related injury [45].

Damage to glomerular vessels can also occur due to anti-phospholipid antibodies present in systemic lupus erythematosus (SLE) that occlude the glomerular vessels and result in chronic inflammation. This chronic inflammation is thought to cause hypertension similar to what is described in the remnant kidney model using ligation. Female NZBWF1 mice are known to produce high titers of antinuclear antibodies. In these mice the kidney is protected from damage by TNF-α blockade [46].

These animal models are all good representations of secondary FSGS in humans. Unfortunately secondary FSGS is only a small part of human FSGS and development to FSGS can often be prevented and/or delayed by treatment of these underlying causes.

### Drug-induced

Adriamycin, puromycin, and streptozotocin are the drugs mostly used to induce FSGS. Additionally, available literature describes a small number of studies conducted with cyclosporine [47] and growth hormone [48], which will not be discussed here.

Most rat strains are susceptible to FSGS induced by adriamycin or puromycin. Most mice strains are not, except for balb/c mice, which are susceptible to adriamycin induced FSGS [28].

Adriamycin is known as an oncolytic antibiotic that can induce proteinuria from the second infusion onward, when given intravenously in rats at 2 mg/kg in a 3-week interval. After 16 weeks, segmental glomerulosclerosis is observed with progression to global glomerulosclerosis and tubulointerstitial fibrosis at 24 weeks. Due to increased serum urea levels, some of the animals will not survive beyond 28 weeks. When given in a single intravenous dose of 5 mg/kg, adriamycin causes sclerosis within 6 months in 50% of animals [49]. Studies that will be discussed used male Munich-Wister rats and injected a single dose. The given doses range from 1,5 to 5 mg/kg in rats [49,50] and 10 to 15 mg/kg in mice [51]. It is important to test the dose before conducting experiments since adriamycin has a small pharmaceutical range, outside of which it becomes toxic. In addition, batch differences can be observed [50].

Puromycin is an antibiotic that inhibits protein synthesis. Puromycin can be given by multiple intraperitoneal injections with initial administration of 10 mg/kg followed by 40 mg/kg every 4 weeks or as a single intravenous dose of 50 mg/kg to cause puromycin aminonucleoside-induced nephrosis (PAN). After injection, rats show an early nephrotic phase peaking at 10 days with complete foot process-effacement followed by apparent resolution. Between 10 and 13 weeks, progressive lower-level proteinuria develops with early segmental sclerotic lesions leading to well-defined segmental sclerosis at 18 weeks [52].

Both adriamycin and puromycin are used frequently to induce FSGS because of their strong dose–response effects [28]. These drugs are often used in the same study in two separate arms. These models have been used to study serial micropuncture analysis of a single nephron while glomerulosclerosis is developing [53]. FSGS treatment studies for which adriamycin and puromycin animal models are used show that the combination of Angiotensin converting enzyme-inhibitors (ACE-I) and Ang II blockers do not have a better effect than ACE-I alone [54]. In addition, they show that MAPK is essential for podocyte injury making p38 MAPK a potential therapeutic target [55] and that vaccination with CCL2 DNA protects against kidney injury after adriamycin injections [56]. Possible new biomarkers for initiation and severity of FSGS, such as fibronectin [57] and Rab-23 [58] respectively, were studied in these animal models as well. Serum fibronectin levels can show a slight but significant increase 3 days before the occurrence of glomerular fibronectin deposits making it a non-specific biomarker for predisposition of FSGS [57]. In case of Rab-23, an autocrine signaling pathway is observed in mesangial cells while developing FSGS, which leads to elevated urine levels of Rab-23 and suppresses this pathway. Therefore, as a biomarker, Rab-23 urine levels may perhaps indicate the severity of FSGS [58].

Both drugs cause direct toxic damage to the podocytes, increase the permeability of glomerular endothelial cells for larger molecules, and reduce glomerular charge selectivity, which leads to tubulointerstitial injury [50]. Since these pathways are different than those known in human FSGS, the relevance of these models is unclear.

Streptozotocin is a naturally occurring chemical, which is toxic to insulin-producing beta-cells of the pancreas. It can be used to treat cancers of the Islets of Langerhans [59] and in medical research to induce diabetes in animal models [60]. The diabetic nephropathy induced in this model precedes the development to FSGS. Intraperitoneal injection of 40 mg/kg in male Syrian APA hamsters induces an ongoing hyperglycemia and hyperlipidemia with high glucose urine levels, which results in glomerular lipidosis after 1 month. After 3 months, FSGS with mesangial expansion is seen. This is caused by an increase of basement membrane-like material, lipid droplets and foam cells. Especially the hyperlipidemia is crucial in this development since it forms the lipid droplets [61].

Studies using streptozotocin-induced hyperglycemia in male Munich Wistar rats show that doxazin, a blood pressure lowering agent, reduces albuminuria by 80%, but does not have an effect on mesangial expansion or progression to glomerulosclerosis. In contrast, proper glycemic control prevents all three [60]. Altered gene expression in the early phase of kidney disease caused by hyperglycemia may be critical in these animals [62].

### Virus-induced

Virus induced animal models that are most often used in FSGS research are HIV-1 based models, in which transgenic mice express HIV-1 accessory genes such as Vpr [63]. These transgenic mice are obtained either by transfecting fertilized eggs of a hybrid between C57BL/6 and DBA/2 with Vpr and the nephrin gene promoter [63] or by using the Tg26 mouse line [64]. In addition, rhesus macaques infected with SIVmacR71/17E, a cloned lymphocyte tropic simian immunodeficiency virus (SIVAN), are used to study FSGS [65]. As mentioned above the virus can inflict damage on podocytes, either by direct infection of these cells or by the release of inflammatory cytokines. Furthermore the virus can transfer from infected T-cells to tubular epithelial cells via viral synapses during cell adhesion [66]. Studies using this animal model have demonstrated protection and reversal of glomerulosclerosis by treatment with Fluvastatin [64] and the cyclin-dependent kinase inhibitor CYC202 [67] respectively.

These animal models are important to study HIVAN since human renal cells also express HIV-1 genes. However, HIVAN is a secondary cause of FSGS and does not widen our knowledge of primary FSGS.

### Podocyte targeting models of FSGS

Since podocytes were identified as the major cellular target in FSGS, new animal models were developed. Genes encoding for podocyte-specific proteins were targeted to obtain knockout mouse models for FSGS. Mpv-17 and α -actinin 4 were the genes targeted most frequently. Podocin deficient mice will be discussed, as well as depletion of podocytes by Thy-1.1 antibody and diphtheria toxin.

Mpv-17 inactivation by retroviral insertion results in foot process flattening and proteinuria within 30 days postpartum, caused by an excessive production of oxygen radicals, and accumulation of lipid peroxidation adducts. After 9–12 months the mice succumb to kidney failure [68].

Studies using Mpv-17 inactivation show depletion of mitochondrial DNA which affects skin, inner ear and kidney. At the onset of FSGS, hardly any mitochondrial DNA is left in the cells of the glomerular tuft [69].

The α-actinin 4 gene encodes for the production of an actin cross-linking protein. Point mutations in this gene cause an autosomal dominant form of human FSGS. There is significant reduction of mRNA and nephrin, a component of the slit diaphragm. The result is a rapidly degrading and deregulated actin cytoskeleton (caused by α-actinin-4) and deterioration of the slit diaphragm (caused by nephrin), leading to early development of proteinuria and FSGS [70]. In studies with α-actinin 4 mutated mice, samples are used for comparison with the autosomal dominant form of human FSGS caused by the same α-actinin 4 mutation [70].

Podocin is encoded by the NPHS2 gene. Mutations in this gene cause familial and sporadic forms of steroid-resistant nephrotic syndrome and FSGS in humans. NPHS2 knockout mice do not develop FSGS, but diffuse mesangial sclerosis. These mice die within days to weeks after birth from renal failure [71]. However, when podocin is inactivated in adult mice by using Cre-loxP technology, it results in nephritic syndrome and FSGS within 4 weeks. This is followed by diffuse glomerulosclerosis and tubulointerstitial injury [72].

An inducible model for FSGS has been generated by introducing the expression of the Thy-1.1 antigen on podocytes. Thy-1.1 is not expressed on podocytes in normal mice. The mouse model was developed by injecting human-mouse Thy-1.1 in zygotes of Thy-1.2 CBA x C57BI mice. After injecting anti-Thy-1.1 monoclonal antibodies, podocytes and parietal epithelial cells (PEC) are damaged leading to podocyte hypertrophy and extracellular matrix production by PEC [73]. Acute albuminuria is induced within a day, and is accompanied by a rapidly developing focal glomerulosclerosis at day 21. The Thy-1.1 transgenic mouse model is appropriate to specifically study the relation between podocyte injury, albuminuria and FSGS development, since it has been proven that in this model the severity of FSGS correlates with the extend of podocyte injury [74]. In this model, it has also been demonstrated that ACE-I is important in preventing development of FSGS, possibly through PEC proliferation blockage [75].

Inducing FSGS in transgenic animals via injection of podocyte-specific toxins was also accomplished by developing rats that express human diphtheria toxin receptors (hDTR) on podocytes. Fertilized Fisher rats were injected with podocin promoter/hDTR to develop these transgenic mice. After reaching adult age, the rats were injected with diphtheria toxin (DT, 1 ml/10 g) causing depletion of podocytes that transport DT into their cytoplasm within 7 days. When 20% of podocytes are lost, mesangial expansion and mild proteinuria develop without loss of kidney function, which suggests that a compensatory mechanism is induced. After depletion of 40% of the podocytes, synechia formation, moderate proteinuria and FSGS lesions including GBM adhesions, PEC migration and ECM formation start to develop. When more than 40% of podocytes are depleted, global sclerosis develops [2].

These models all affect podocytes, either by targeting existing genes and their encoding proteins, or by the transfection of specific receptors on podocytes, which can specifically be targeted. The models using existing genes cover less than 8% of human causes for FSGS. Both podocyte depletion models give important information about the continuous progression of FSGS in a dose dependent manner, but do not address the cause of primary FSGS.

### Circulating permeability factors

Animal studies have helped to prove the existence of circulating permeability factors causal for FSGS by showing that FSGS can be induced in rats after injection with serum of FSGS patients. Both studies discussed here used Sprague–Dawley rats that were injected with serum of biopsy-proven FSGS in patients with primary disease. These studies show that a single injection of FSGS patient serum causes transient albuminuria and proteinuria in rats [77] and that especially serum from patients with the collapsing FSGS variant leads to glomerular tuft retraction and podocyte damage [76].

To date, there is no consensus on which candidate factor the actual “FSGS-factor” is or where it is produced. These studies support the existence of a circulating permeability factor, and have the potential to identify this “FSGS-inducing factor”.

### Spontaneously developing FSGS

In the literature, only one spontaneously developing FSGS mouse model has been published. Studies using this FGS/Nga mouse model have appeared between 1991 and 2004. The mouse model was established after interstrain crossbreeding of CBA/Nga and RFM/Nga offspring. The strain spontaneously developed FSGS lesions at 3 months and severe glomerulosclerosis within one year. Studies of this mouse model revealed dense deposits in the mesangium containing IgA, IgM, C3 and the retroviral envelope antigen. Breeding of these animals was possible up to 18 generations [78].

A study using this mouse model showed that bone marrow transplantation (BMT) from normal mice to FSGS mice ameliorates FSGS and that BMT or transfer of purified hematopoietic stem cells from FSGS mice to normal mice induced FSGS [79]. A study was conducted to locate quantitative trait loci (QTL) affecting the glomerulosclerosis index (GSI) in these mice. Two QTL were found on chromosomes 8 and 10. The presence of Gsi1 increased the GSI while the presence of Gsi2 decreased GSI [80].

Currently, only some embryos of this mouse model are left in Japan, but no active research seems to be performed [78] (Table 1).

**Table 1:** Overview of animal models discussed in this review

**Animal models**	**Method of developing**	**Examples of FSGS research conducted using these animal models**
Remnant kidney model [28-34]	5/6 renal mass resection	Use of thromboxane inhibitors [35]
		Use of lipid lowering agents [36]
		Use of peroxisome proliferator [37]
		Use of antifibrotic agents [38]
		Absence of p21 [39]
		Absence of Apolipoprotein E [40]
Renal mass reduction due to systemic disease [41,44]	Salt sensitive animals	Role of hypertension [41,42]
	Norepinephrine	
	Angiotensin	
	Zucker rats	Role of macrophage influx [43]
	Munich Wistar rats	Use of Endothelin-1 inhibition [44,45]
	SLE	Use of TNF-α blockade [46]
Drug-induced [28,49-52]	Adriamycin Puromycin	Use of ACE-I + Ang II inhibitors [54]
		Role of MAPK [55]
		Use of CCL2 vaccination [56]
		Role of fibronectin as biomarker [57]
		Role of Rab 23 as biomarker [58]
	Streptozotocin	Role of good glycemic versus blood pressure control [60]
Virus-induced [63-65]	Vpr-gene	Use of fluvastatin [64]
		Use of CYC202 [67]
	SIVAN	[65]
Genetic targets [2,68,70,71,73]	Mpv-17	Role of mitochondrial DNA [69]
	α-actinin 4	Genetic human FSGS comparison [70]
	Podocin-deficiency	Knockout versus depletion [71,72]
	Thy-1.	Role of ACE-I [75]
	hDTR	Presence of threshold [2]
Circulating permeability factors [76,77]	Collapsing variant serum injection	Podocyte damage after injection [76]
	Supernatant injection	Induction of transient proteinuria [77]
Spontaneously [78]	Accidental	Use of BMT [79]
		Role of QTL [80]

Abbreviations: SLE, Systemic lupus erythematosus; TNF- α, Tumor necrosis factor-alfa; ACE-I, Angiotensin-converting-enzyme inhibitor; Ang II, Angiotensin II; MAPK, Mitogen-activated protein kinases; CCL2, Chemokine (C-C motif) ligand 2; Vpr, Viral protein R; SIVAN, Simian immunodeficiency virus associated nephropathy; DNA, Deoxyribonucleic acid; FSGS, Focal segmental glomerulosclerosis; hDTR, human diphtheria toxin receptor; BMT, Bone marrow transplantation; QTL, Quantitative trait loci.

## Review and conclusions

Much of our knowledge on FSGS has come from a variety of animal models. However, to date, there is still no exact understanding of the pathogenesis of idiopathic FSGS, and there is no definite curative treatment. Therefore, more research on FSGS is necessary. Although kidney transplantation is currently the best treatment option, the donor shortage, the high posttransplant FSGS recurrence rate, and the side effects of immunosuppression, all warrant improvement of treatment. Our inability to fully understand the pathogenesis and find curative treatment for FSGS may be due to the fact that almost all of the animal models used are based on the induction of secondary forms of FSGS. Most of them show an acute onset of proteinuria and FSGS, whereas in human FSGS 80% is idiopathic and develops as a chronic disease over time. Future FSGS research requires an animal model that resembles human primary FSGS in that it has a spontaneous onset and shows slow chronic deterioration. Such a model is currently lacking.

## Abbreviations

FSGS: Focal segmental glomerulosclerosis; ESRD: End-stage renal disease; KT: Kidney transplantation; HIV: Human immunodeficiency virus; CD2AP: CD2-associated protein; TRPC6: Transient receptor potential cation channel 6; PLCε1: Phospholipase C ε1; GTP-ase: Guanosine triphosphatase; INF 2: Inverted formin 2; MYH9: Myosin, heavy chain 9; APOL1: Apolipoprotein L1; SuPAR: Soluble urokinase-type plasminogen activator receptor; GBM: Glomerular basement membrane; Ang II: Angiotensin II; TGF-β: Transforming growth factor beta; VEGF: vascular endothelial growth factor; IFN: Interferon; MHC II: Major histocompatibility complex; HIVAN: HIV-associated nephropathy; Nef: Negative regulatory factor; Vpr: Viral protein R; PEC: Parietal epithelial cells; ECM: Extracellular matrix; NaCl: Sodium Chloride; NE: Norepinephrine; SLE: Systemic lupus erythematosus; TNF- α: Tumor necrosis factor-alfa; PAN: Puromycin aminonucleoside-induced nephrosis; H2O2: Hydrogen peroxide; ACE-i: Angiotensin-converting-enzyme inhibitor; MAPK: Mitogen-activated protein kinases; CCL2: Chemokine (C-C motif) ligand 2; DNA: Deoxyribonucleic acid; SIVAN: Simian immunodeficiency virus associated nephropathy; mRNA: Messenger ribonucleic acid; hDTR: Human diphtheria toxin receptors; DT: Diphtheria toxin; IgA: Immunoglobulin A; IgM: Immunoglobulin M; C3: Complement component 3; BMT: Bone marrow transplantation; QTL: Quantitative trait loci; GSI: Glomerulosclerosis index.

## Competing interests

Regarding this review on used animal models for FSGS, we declare to have no financial disclosures.

## Authors’ contributions

SM drafted the manuscript. FD, MH and RB revised and approved the final manuscript. All authors read and approved the final manuscript.

## Pre-publication history

The pre-publication history for this paper can be accessed here:

http://www.biomedcentral.com/1471-2369/14/74/prepub
